# From homogeneity to heterogeneity: Refining stochastic simulations of gene regulation

**DOI:** 10.1016/j.csbj.2025.01.004

**Published:** 2025-01-15

**Authors:** Seok Joo Chae, Seolah Shin, Kangmin Lee, Seunggyu Lee, Jae Kyoung Kim

**Affiliations:** a Department of Mathematical Sciences, KAIST, Daejeon, 34141, Republic of Korea; b Biomedical Mathematics group, Pioneer Research Center for Mathematical and Computational Sciences, Institute for Basic Science, Daejeon, 34126, Republic of Korea; c Department of Bioengineering, Rice University, Houston, 77005, TX, United States of America; d Department of Applied Mathematics, Korea University, Seoul, 02841, Republic of Korea; e Department of Medicine, College of Medicine, Korea University, Seoul, 02841, Republic of Korea

## Abstract

Cellular processes are intricately controlled through gene regulation, which is significantly influenced by intrinsic noise due to the small number of molecules involved. The Gillespie algorithm, a widely used stochastic simulation method, is pervasively employed to model these systems. However, this algorithm typically assumes that DNA is homogeneously distributed throughout the nucleus, which is not realistic. In this study, we evaluated whether stochastic simulations based on the assumption of spatial homogeneity can accurately capture the dynamics of gene regulation. Our findings indicate that when transcription factors diffuse slowly, these simulations fail to accurately capture gene expression, highlighting the necessity to account for spatial heterogeneity. However, incorporating spatial heterogeneity considerably increases computational time. To address this, we explored various stochastic quasi-steady-state approximations (QSSAs) that simplify the model and reduce simulation time. While both the stochastic total quasi-steady state approximation (stQSSA) and the stochastic low-state quasi-steady-state approximation (slQSSA) reduced simulation time, only the slQSSA provided an accurate model reduction. Our study underscores the importance of utilizing appropriate methods for efficient and accurate stochastic simulations of gene regulatory dynamics, especially when incorporating spatial heterogeneity.

## Graphical abstract

**Figure:**
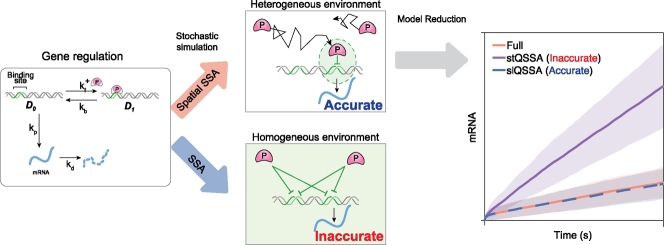

## 1 Introduction

Gene regulation is a fundamental process that governs cellular functions, organismal development, and responses to environmental changes [1], [2], [3]. In eukaryotes, this process is primarily regulated by interactions between DNA and DNA-binding proteins (e.g., transcription factors) within the nucleus [3], [4], [5], [6]. The molecules involved in gene regulation are often present in limited quantities, typically consisting of one or two DNA copies and transcription factor numbers of less than a hundred [7]. Consequently, the stochastic nature of gene regulation plays a critical role. This stochasticity leads to fluctuations in gene expression, which can be crucial for processes such as cell division and environmental adaptation. To comprehend the impact of stochasticity on gene regulation, the chemical master equation (CME), which describes the probabilities of different molecular states over time, has been frequently used. However, analytical solutions to the CME are typically challenging to obtain [8], [9], [10], [11], [12], [13], [14]. To address this, the stochastic simulation algorithm (SSA) [15], represented by the Gillespie algorithm, has been widely used for simulating gene regulation [16], [17], [18], [19], [20], [21], [22], [23], [24], [25], [26], [27].

The SSA assumes homogeneity within the nucleus, treating molecules as evenly distributed. However, in reality, genes are localized at specific locations within the nucleus rather than being uniformly distributed [28], [29]. This spatial heterogeneity may affect the accuracy of the SSA in describing actual transcription processes. The impact of spatial heterogeneity is expected to be minimal when diffusion of proteins occurs rapidly. However, under slow diffusion conditions, assuming a homogeneous distribution can lead to significant deviations from reality.

When the SSA does not accurately capture the effects of spatial heterogeneity, it is crucial to employ alternative stochastic simulation methods that incorporate spatial heterogeneity. Previous studies have addressed spatial heterogeneity using either compartment-based or agent-based methods. Compartment-based methods, such as compartment-based SSA [30], URDME [31], MesoRD [32], SmartCell [33], and Lattice Microbes [34], divide the domain into small compartments where diffusion and reactions are modeled using the SSA or its variants. On the other hand, agent-based methods like STEPS [35] and Smoldyn [36] simulate the Brownian motion of individual molecules and reactions that occur upon molecular collisions. By incorporating spatial heterogeneity, these methods have effectively described complex biological systems [37], [38], [39], [40], [41], [42], [43], [44], [45].

Despite accurate descriptions of systems, stochastic simulations become computationally intensive when spatial heterogeneity is incorporated [46], [47]. To reduce this computational burden, the Langevin approach, which simplifies the SSA, has recently been proposed for application to spatial SSA [48], [49], [50]. However, this method may not be suitable for systems describing gene regulation as it typically requires a large number of molecules. For such systems, the quasi-steady-state approximation (QSSA) presents a promising alternative. Various QSSA methods have successfully accelerated the SSA by simplifying gene regulation models [8], [9], [10], [11], [12], [13], [51], [52], [53], [54], [55], [56], [57], [58], [59], [60], [61]. However, it remains unclear whether QSSA can be effectively applied to the spatial SSA.

In this study, we evaluated the accuracy of the SSA in describing gene regulation within heterogeneous environments by comparing the standard SSA with the spatial SSA. To simulate the spatial SSA, we employed the compartment-based SSA [30], since the agent-based SSA can be infeasible for a large number of molecules, whereas the compartment-based approach remains feasible [62], [63]. Our findings indicate that the SSA and the spatial SSA produce different outcomes when transcription factors diffuse slowly. Furthermore, when these transcription factors are non-uniformly distributed in the nucleus or when there are multiple DNA binding sites, this difference becomes larger. Additionally, we examined whether model reduction techniques for the SSA can be extended to the spatial SSA. We found that using the stochastic total quasi-steady-state approximation (stQSSA) to reduce the gene regulation model is accurate for the SSA but yields significant errors for the spatial SSA. On the other hand, the reduced model using the stochastic low-state quasi-steady-state approximation (slQSSA) provides accurate simulations for both the SSA and the spatial SSA, particularly when gene copy numbers are small. Our study provides guidelines on how to simulate and simplify models describing gene regulation by transcription factors with slow diffusion in spatially heterogeneous environments.

## 2 Results

### 2.1 Gene regulation models can be simulated with SSA or spatial SSA

Transcriptional regulation is a key mechanism of gene expression, often involving transcription factors binding to DNA [3], [4], [5], [6]. These factors can either promote or inhibit transcription. Our study investigates a simplified gene regulatory network based on a well-established model that describes gene expression modulated by transcription factor binding [26], [64], [65].

**Fig. 1:**
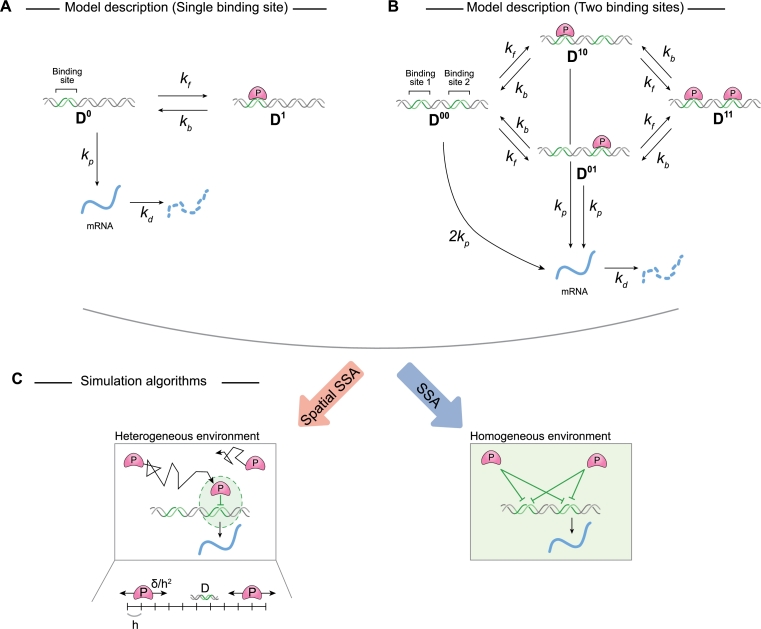
**Frameworks for stochastic simulation of gene regulation with and without spatial heterogeneity.** (A) Schematic diagram of the model describing the repressor protein (P) to a binding site on DNA. P can reversibly bind to an unoccupied site on the DNA (D_0_) with a binding rate *k*_*f*_ and an unbinding rate *k*_*b*_. Unoccupied DNA (D_0_) is transcribed at a rate of *k*_*p*_. When the binding site is occupied (D_1_), the transcription is repressed. Transcribed mRNA decays at the rate of *k*_*d*_. (B) When the DNA has two binding sites, P can reversibly bind to either site. If both binding sites are unoccupied (D_00_), mRNA is transcribed with the rate of 2*k*_*p*_. If one site is occupied (D_01_ or D_10_), the transcription rate is halved. P can still reversibly bind to the other site with the same binding rates (*k*_*f*_), indicating that the binding of P to each site occurs independently. When both sites are occupied (D_11_), transcription is fully repressed. (C) These models can be simulated using either the spatial SSA (left) or the SSA (right). The spatial SSA describes not only the reactions but also the diffusion of P (left). To describe diffusion, we divided the domain into multiple compartments of size *h*. The diffusion rate is calculated by *δ*/*h*^2^, where *δ* is the diffusion coefficient of P. P is required to diffuse to DNA before binding, ensuring that only P in proximity to DNA binding sites can bind. In contrast, the SSA assumes a homogeneous environment for DNA and P, allowing P to bind to DNA binding sites from anywhere in the nucleus (right).

As a basic case, we modeled gene regulation of DNA with a single binding site (Fig. 1A). In this model, the DNA ($D^{0}$) has one binding site that can reversibly bind to a repressor protein (P) with a binding rate of $k_{f}$ and an unbinding rate of $k_{b}$. When the binding site is occupied ($D^{1}$), transcription is inhibited, but when the site is unoccupied, transcription proceeds at a rate of $k_{p}$. The mRNA that is produced subsequently degrades at a rate of $k_{d}$.

In gene regulation, it is common for DNA to contain multiple binding sites for transcription factors [66], [67], [68], [69], [70]. To describe gene regulation involving multiple binding sites, we first examined a simple model with DNA containing two binding sites (Fig. 1B and Eq. (1)). Following previous studies [71], [72], we assumed that each binding site independently influences transcription. Specifically, when the DNA is unoccupied ($D^{00}$), transcription occurs at a rate of $2k_{p}$. If either binding site is occupied ($D^{01}$ or $D^{10}$), the transcription rate is halved. P can bind to the remaining site, and when both binding sites are occupied ($D^{11}$), transcription is fully suppressed. Since the model distinguishes all DNA binding statuses, simulation and analysis can be complex. Given this complexity, we sought a way to simplify the model while maintaining its essential dynamics. To achieve this, we leveraged the fact that the transcription rate is proportional to the number of free binding sites. For example, mRNA is transcribed from $D^{00}$, which contains two free binding sites, at a rate of $2k_{p}$. Similarly, mRNA is transcribed at a rate of $k_{p}$ from $D^{01}$ and $D^{10}$. Thus, by introducing *D*, which is the number of unoccupied binding sites, we can describe the transcription from various statuses of DNA ($D^{00},D^{01},D^{10}$, and $D^{11}$) by simply using $k_{p}D$. That is, we can describe numerous transcription reactions from various DNA binding statuses with a single reaction from D. This approach allows us to construct a simpler model (Eq. (4)) with dynamics identical to the original (Eq. (1)) (see Methods for details). Consistent with this notation, we use *P* and D:P to represent the amount of protein (P) and protein-bound binding sites (D:P), respectively.

To simulate gene regulation models, two methods can be utilized: the spatial SSA and the SSA (Fig. 1C). The spatial SSA, based on the compartment-based Gillespie algorithm [30], describes the diffusion and reactions of species. Specifically, the spatial SSA divides the domain into compartments of size *h*, and P can diffuse across compartments at a rate of $\delta /h^{2}$, where *δ* is the diffusion coefficient of P. The binding of D and P occurs only when both are in the same compartment. While the spatial SSA provides a detailed simulation, it can be computationally intensive. To circumvent this, previous studies employed the Gillespie algorithm to implement the SSA for computational efficiency. The SSA assumes a homogeneous environment where all P can bind to the sites with the same propensity, ignoring spatial heterogeneity. While this simplifies the simulation and speeds it up, using the SSA may capture the gene regulation inaccurately when spatial heterogeneity plays a role.

### 2.2 Utilizing SSA results in an error in simulating gene regulation models when the diffusion of transcription factors is slow

While the SSA assumes that a nucleus is a well-mixed environment, this is a biologically irrelevant assumption since DNA is localized in a specific location. To investigate whether the SSA can still accurately capture transcription, we compared the SSA with the spatial SSA. By simulating the gene regulation model (Fig. 1) with the SSA and the spatial SSA, we quantitatively compared mRNA levels over time under various initial conditions. Specifically, we compared the mean and standard deviation of mRNA amounts across 1,000 simulation iterations for each method.

**Fig. 2:**
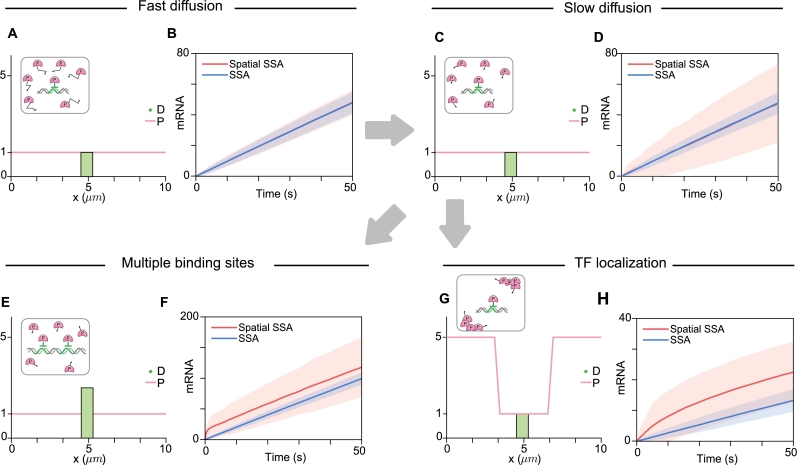
**Utilizing the spatial SSA is essential for accurately simulating gene regulation with multiple binding sites or the non-uniform distribution of transcription factors within the nucleus.** (A) The regulation of DNA with a single binding site, when P is uniformly distributed, is described by the initial condition where *P* = 1 throughout the nucleus and *D* = 1 at the nucleus center. The diffusion coefficient of P was set to $20\;{\mu \text{m}}^{2}/s$, which lies in the typical range of protein diffusion coefficients. (B) With these initial conditions, both the spatial SSA and the SSA result in the same average mRNA production and the same standard deviation of mRNA production over time. (C) To describe the scenario when diffusion occurs slowly, the same initial conditions as in (A) were used, but the diffusion coefficient of P was decreased to $0.2\;{\mu \text{m}}^{2}/s$. (D) When diffusion occurs slowly, the SSA results in the same average mRNA production as the spatial SSA; however, the SSA underestimates the standard deviation of the mRNA production. This occurs because diffusion of *P* to *D* results in additional noise in the transcription. (E) To describe the regulation of DNA with two binding sites when *P* is uniformly distributed, we utilized the initial condition where *P* = 1 throughout the nucleus and *D* = 2 at the nucleus center. (F) With these initial conditions (E), the SSA underestimates the average mRNA production more than the spatial SSA does. This is because the SSA allows transcription factors at every location to bind to the DNA, which results in an overestimation of the number of occupied sites. (G) To describe when transcription factors are localized in a specific position, we utilized a non-uniform distribution. Specifically, *P* is assumed to be accumulated (*P* = 5) at the periphery of the nucleus, while *P* = 1 is assumed to be near the nucleus center. (H) With these initial conditions, the SSA highly underestimates the mRNA production compared to the spatial SSA.

We first simulated from a simple model where D is localized in the center of the nucleus and P is initially uniformly distributed across the nucleus (Fig. 2A). When diffusion is fast, the SSA and the spatial SSA produce similar means and standard deviations of mRNA amounts (Fig. 2B). This situation resembles well-mixed conditions, resulting in no significant differences between the SSA and the spatial SSA.

When diffusion is slow, the SSA underestimates the variance in mRNA levels while accurately predicting the average mRNA production compared to the spatial SSA (Fig. 2C-D). Slow diffusion restricts transitions between active and inactive DNA states, as P moves more slowly into and out of DNA-containing compartments. This leads to prolonged periods of either high mRNA production (active DNA) or no change in mRNA levels (inactive DNA), resulting in greater variability in the mRNA amounts predicted by the spatial SSA under these conditions (Fig. 2D). This difference in mRNA variance decreases as the diffusion becomes faster. Specifically, when the diffusion coefficient reaches approximately 20 ${\mu \text{m}}^{2}/s$, where the diffusion timescale for movement across a unit distance (${\left(1\;\mu \text{m}\right)}^{2}/\delta =0.05s$) matches the transcription timescale ($1/k_{p}=0.02s$), the standard deviation of mRNA production is higher in the spatial SSA compared to the SSA (Supplementary Fig. S1). Despite these differences in variance, the mean mRNA production remains unaffected by the diffusion coefficient, as P remains uniformly distributed on average (Fig. 2D). Consequently, the SSA, which assumes rapid diffusion of proteins, aligns closely with the average mRNA production predicted by the spatial SSA but fails to capture the increased variance under slow diffusion conditions.

We further investigated whether the SSA can accurately describe gene regulation with multiple binding sites. We used an initial condition where two binding sites are located in the center of the nucleus (Fig. 2E). In this case, the SSA underestimates both the average and the variance of mRNA transcription compared to the spatial SSA (Fig. 2F). This discrepancy arises because the SSA does not account for the time it takes for P to diffuse and reach the DNA binding sites. When the local concentration of P is insufficient to saturate all the binding sites, the SSA inaccurately assumes that every P molecule can bind to D regardless of its spatial position. However, in reality, only P molecules close to D can bind. Consequently, the SSA model shows DNA becoming occupied more rapidly than the spatial SSA model does, resulting in greater inhibition. Additionally, when the distribution of P is nonuniform, such that the local concentration of P near DNA is lower than the average concentration, the SSA underestimates mRNA transcription compared to the spatial SSA (Fig. 2G-H). This underestimation of transcription occurs because the SSA overestimates the amount of P, thereby overestimating the repression of D.

While our investigation primarily focused on inhibitory gene regulation, it is important to recognize that gene regulation can also be activating, where the binding of a protein to DNA induces transcription. To include activating gene regulation in our study, we modified our model (Eq. (4)) to simulate mRNA (M) production from a protein-bound binding site (D:P) instead of from unbound binding sites (D). We then compared the spatial SSA to the SSA under various conditions. Similar to inhibitory gene regulation, slower diffusion rates led to an overestimation of the variance in mRNA levels and even altered mean mRNA levels when the protein distribution changed (Supplementary Fig. S2). Consequently, using the SSA to describe gene regulation under conditions of slow diffusion, multiple binding sites, and non-uniform distribution of transcription factors can lead to results that differ significantly from those observed in real biological systems.

### 2.3 Stochastic tQSSA can lead to an erroneous reduction of spatiotemporal models

In the previous section, we demonstrated the necessity of the spatial SSA to model gene regulation accurately under slow diffusion. Under these conditions, the majority of computation time is spent simulating fast reactions. Since reversible binding between a transcription factor and DNA typically occurs faster than transcription, these reversible binding reactions become the primary source of computational cost. To address this, we aimed to develop a more efficient method to reduce the spatial SSA by eliminating fast reactions without compromising accuracy.

Accurate methods for eliminating fast reactions in the SSA have been previously reported [8], [9], [10], [11], [12], [13], [51], [52], [53], [54], [55], [56], [57], [58], [59], [60], [61]. These methods are based on the assumption that species involved in rapid binding and unbinding reactions reach their quasi-steady states (QSSs) faster than other species not affected by reversible binding. This assumption allows for replacing the variables involved in fast reactions with their quasi-steady-state approximations (QSSAs), thereby eliminating the fast reactions. However, since these methods are derived for the SSA under the assumption of homogeneous environments, it is unclear whether QSSAs remain accurate in heterogeneous environments. Therefore, in this section, we investigated the applicability of QSSAs derived for the SSA within the spatial SSA, particularly under conditions of slow diffusion.

**Fig. 3:**
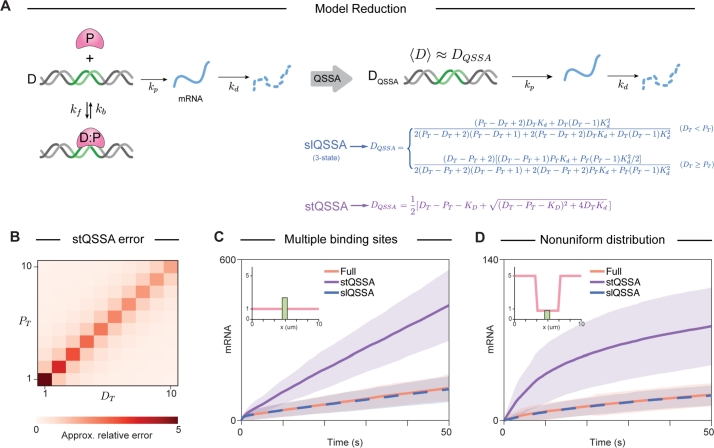
**slQSSA, but not stQSSA, provides an accurate reduction of the full model describing gene regulation.** (A) The full model describing gene regulation, based on mass-action kinetics, can be reduced by replacing *D* by its stQSSA or slQSSA. Both approximations assume that *D* reaches a QSS rapidly, which is determined by the total DNA amount (*D*_*T*_) and the total protein amount (*P*_*T*_). See Methods for details. (B) The relative error of the stQSSA increases as the sensitivity of the *D*_*QSSA*_ increases. The stQSSA results in a large sensitivity, and thus a large error when *D*_*T*_ ≈ *P*_*T*_. (C-D) As a result, stQSSA overestimates the average mRNA production in both cases: when a small number of transcription factors interact with the two binding sites (C, inset), and when *D*_*T*_ ≈ *P*_*T*_ due to *P* localization at the nucleus periphery (D, inset). In contrast, slQSSA accurately estimates the average mRNA production within the nucleus, regardless of the initial conditions. For C and D, the diffusion coefficient of P was set to 0.2 ${\mu \text{m}}^{2}/s$.

The QSSAs of species in homogeneous environments are obtained assuming the QSS of binding and unbinding reactions. For stochastic models, the QSSAs for species are obtained as the stationary average number of the species when the system reaches QSS. In our model, we calculate the QSSA of the number of free binding sites ($D_{QSSA}$) as it directly affects the amount of mRNA. Calculating the $D_{QSSA}$ involves solving the CMEs governing gene regulation, which includes all reactions and diffusion processes and cannot be solved analytically. However, an analytical solution is possible for the reduced CME that accounts solely for protein binding and unbinding at binding sites, particularly when the DNA has a small number of binding sites. This reduction is valid when binding and unbinding reactions occur significantly faster than other processes, enabling an accurate approximation of the full model. This is called the stochastic low-state QSSA (slQSSA) (Fig. 3A) [13]. The slQSSA becomes highly complex and difficult to analyze as the number of binding sites increases. In such cases, the stochastic total QSSA (stQSSA) [9], [10], [11], [12], [13], [52], [54], [55], [56], [57], [58], [59], [60], [61] can be used as an alternative. The stQSSA of the number of binding sites, *D*, is a non-elementary function, which is obtained from the steady-state solution of the associated differential equation in terms of the total variables, $D_{T}=D+D:P$ [9], [10], [11], [12], [13], [52], [54], [55], [56], [57], [58], [59], [60], [61], where $D_{T}$ represent the number of total binding sites, respectively. This is then used to calculate propensity functions in the Gillespie algorithm for stochastic simulations. Although the stQSSA is generally accurate in the SSA [9], [10], [11], [12], [13], [52], [54], [55], [56], [57], [58], [59], [60], [61], it has been reported that tight binding with similar amounts of *D* and *P* results in discrepancies between the predicted and actual average free DNA [13]. Specifically, the upper bound of the relative error in the stQSSA increases 1) when the dissociation constant $K_{d}=k_{f}/k_{b}\cdot \Omega$ becomes significantly less than 1, where Ω denotes the system size, and 2) when $D_{T}\approx P_{T}$, with $P_{T}=P+D:P$. Thus, the relative error in the stQSSA increases as the absolute value of $D_{T}-P_{T}-K_{D}$ decreases [13]. Consequently, when the molar ratio of *D* and *P* is 1:1 and the binding is tight ($K_{d}<<1$), the error upper bound reaches its maximum (Fig. 3B) [13].

We examined the accuracy of two QSSAs, the slQSSA and the stQSSA, derived for the SSA within the spatial SSA. Specifically, we compared the mRNA levels obtained from a gene regulation model describing multiple binding sites by simulating the full model, the slQSSA model, and the stQSSA model. We chose initial conditions where the overall molar ratio of D and P deviates from 1:1, a scenario in which the stQSSA is expected to perform well (Fig. 3C, inset).

Unexpectedly, the stQSSA model overestimated the mRNA amount compared to the full model (Fig. 3C). This overestimation occurred because the local ratio of *D* and *P* near the DNA approached 1:1, leading to an overestimation of *D*. In contrast, the slQSSA captured the full model accurately (Fig. 3C). When we repeated the simulation with initial conditions describing a non-uniform distribution of *P*, the stQSSA again resulted in an error (Fig. 3D). This error was due to the local ratio of *D* and *P* approaching 1:1. However, the slQSSA once again accurately captured the full model (Fig. 3D). Furthermore, the slQSSA significantly reduced simulation time. For the simulation shown in Fig. 3C with 100 iterations, the full model required ∼160 seconds of computational time, whereas the slQSSA model took just ∼10 seconds—an approximately ∼10-fold speedup for the equivalent simulation (Supplementary Fig. S3A). This efficiency gain becomes more pronounced as binding and unbinding rates increase proportionally. Specifically, while proportional increases in these rates led to longer computation times for the full model, the mRNA dynamics remain unchanged under such conditions (Supplementary Fig. S3). In contrast, the slQSSA model did not further increase computational time, since the slQSSA model relies on the dissociation constant rather than individual rate constants (Eq. (6)). Notably, the slQSSA achieved a 40- to 120-fold speedup while preserving stochastic dynamics equivalent to those of the full model (Supplementary Fig. S3).

Taken together, we found that stQSSA can be inaccurate in reducing the full model for spatial SSA, even under conditions previously predicted to ensure accuracy in reducing the full model for SSA. However, even with the spatial SSA, the slQSSA always correctly reduced the full model, as it is the analytic solution of CMEs. These findings suggest that we should avoid using the stQSSA to reduce spatially heterogeneous models.

### 2.4 Stochastic tQSSA can distort oscillatory dynamics

So far, we have shown that the stQSSA does not accurately approximate the full model even when the overall molar ratio of D and P is different from 1:1 by examining cases when the molar ratio is conserved. However, in real cells, the number of transcription factors changes over time, which can cause the molar ratio to vary. This raises the question of whether the stQSSA is accurate in situations where the molar ratio is 1:1 for only a short period. To investigate this, we examined the accuracy of the stQSSA and the slQSSA in a model exhibiting oscillatory dynamics.

**Fig. 4:**
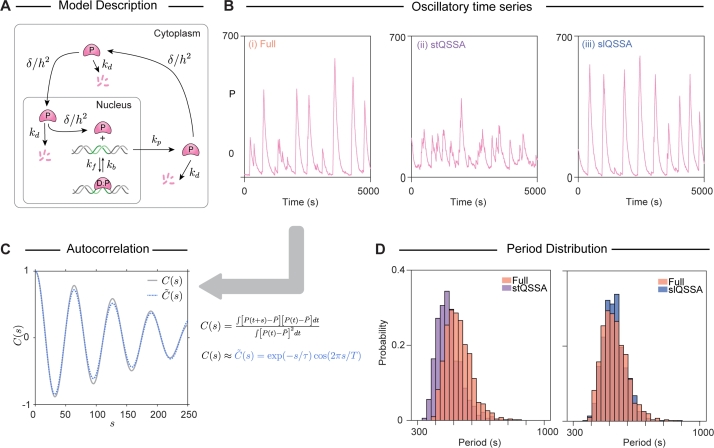
**The stQSSA underestimates the period of the biological oscillation** (A) Full model diagram of an oscillatory transcriptional negative feedback loop. From the unoccupied DNA, the repressor protein (*P*) is produced and then translocated to the periphery of the cytoplasm. Then *P* diffuses to the nucleus with the diffusion coefficient of $0.2\;{\mu \text{m}}^{2}/s$. After entering the nucleus, the *P* diffuses to the DNA and binds to it to form a complex, which is transcriptionally inactive, and thus represses its own production. As the reversible binding between *P* and *D* is rapid, by replacing *D* with its QSSAs (either stQSSA or slQSSA), we can obtain reduced models. (B) Oscillatory trajectories of *P* simulated with the full model (i), the stQSSA model (ii), and the slQSSA model (iii). In the stQSSA model, transcription occurs more frequently than in the full model, while the slQSSA model shows transcription occurring at a similar frequency to that of the full model. (C) To measure the period of each time series, the autocorrelation of the time series (*C*(*s*)) was calculated. The period (*T*) was then estimated by fitting the resulting data to a decaying cosine function $\overset{˜}{C}(s)$. (D) The stQSSA model predicts a shorter period than the full model (left). In contrast, the slQSSA model accurately predicts the period of the full model (right). The distribution of periods was obtained from the 1,000 iterations of the simulation.

To explore this, we constructed a negative feedback model designed to generate oscillatory behavior (Fig. 4A). Specifically, when P is produced, it instantly translocates to the cell periphery and then diffuses to the center of the nucleus, where D is located. Upon binding to D, P represses its own production, leading to a decrease in its amount. As the amount of P decreases and D becomes unbound, transcription resumes, increasing the amount of P. This negative feedback loop induces oscillations through repeated cycles.

To evaluate whether the reduced models can accurately capture the oscillations of the full model, we simulated these models using the spatial SSA (Fig. 4A). The oscillations of *P* in the full model (Fig. 4B(i)) are not accurately captured by the stQSSA (Fig. 4B(ii)). The inaccuracy of the stQSSA model arises because transcription occurs more frequently when the molar ratio is close to 1:1 (Fig. 3C-D), leading to faster repression and a shorter oscillation period. In contrast, the slQSSA model closely captures the oscillations of the full model (Fig. 4B(iii)).

To quantify the period of the time series obtained from each model, we utilized the autocorrelation of each time series [27], [73], [74]. Specifically, we estimated the period by fitting the oscillatory time series to a decaying cosine function (Fig. 4C). The stQSSA model underestimated the oscillation period of the full model, while the slQSSA model accurately predicted the oscillation period (Fig. 4D). This indicates that the stQSSA can distort oscillatory dynamics even if the 1:1 ratio is maintained for only a short time. On the other hand, the slQSSA remains accurate, making it a more reliable choice for properly reducing spatially heterogeneous models.

## 3 Discussion

Stochasticity plays a crucial role in gene regulation due to the limited number of molecules involved in the process [3]. To understand this phenomenon, previous studies have extensively employed the SSA, which assumes the homogeneity of the system [16], [17], [18], [19], [20], [21], [22], [23], [24], [25], [26], [27]. Our study investigated whether this assumption is appropriate for accurately describing biological systems. Our findings reveal that when transcription factors diffuse slowly, the SSA fails to adequately capture spatial heterogeneity within the cellular environment (Fig. 2C-D). Conversely, when the diffusion of transcription factors is rapid compared to reaction rates, the spatial SSA and the SSA yield similar results (Fig. 2A-B). This is because when the diffusion timescale is shorter than the reaction timescale, the system becomes homogenized before reactions affect the system appreciably. Typically, reaction timescales for binding, unbinding, and catalysis are under 1*s* [75]. Given that the size of a eukaryotic nucleus is on the micrometer scale, ranging from 2 to 10 μm [76], and the diffusion coefficient of transcription factors (*δ*) scale ranges from 0.5 to 5 ${\mu \text{m}}^{2}/s$ [76], [77], the diffusion time scale ($={\left(1\;\mu \text{m}\right)}^{2}/\delta$) is estimated to be approximately 0.2 to 2*s*. Consequently, the diffusion timescale of transcription factors can be slower than the reaction timescale. In such cases, employing the spatial SSA becomes crucial for accurately simulating gene regulation.

Stochastic simulations often require significantly more time than deterministic methods, necessitating acceleration techniques. Various QSSA methods, such as the sQSSA, stQSSA, and slQSSA, have been employed to speed up simulations in the SSA [8], [9], [10], [11], [12], [13], [51], [52], [53], [54], [55], [56], [57], [58], [59], [60], [61]; however, a clear understanding of their effectiveness in the spatial SSA has been elusive. Our study evaluated the applicability of these QSSA methods under conditions where transcriptional factors diffuse slowly compared to binding and unbinding reactions. Given the success of the tQSSA in deterministic partial differential equation simulations [78], it seemed a natural candidate for application to stochastic spatial simulations. However, our findings indicate that the stQSSA can introduce substantial errors in heterogeneous environments commonly found in the spatial SSA simulations (Fig. 3). These errors arise because the stQSSA may satisfy the validity conditions globally in the SSA, but not within the confined regions where DNA is localized. In contrast, the slQSSA, derived by analytically solving the CME under the assumption of a small number of DNA binding sites, consistently provides accurate estimates (Fig. 3). Moreover, this accuracy was maintained even in two-dimensional simulations (Supplementary Fig. S4), suggesting that the slQSSA is effective in higher-dimensional domains (e.g., realistic cellular environments), provided that diffusion remains slower than rapid reactions. Taken together, utilizing the slQSSA is recommended to reduce simulation time in spatiotemporal stochastic models.

Our study opens several avenues for future research. The tQSSA has been proposed as an alternative to the well-established Michaelis-Menten equation for simplifying complex models. Specifically, the tQSSA offers more precise estimates of pharmacokinetic parameters such as drug clearance [79], [80], [81]. Notably, most research has focused on deterministic and spatially homogeneous systems; however, our findings indicate that the effectiveness of the tQSSA may vary in the presence of stochasticity and spatial heterogeneity. Investigating the application of the tQSSA in such contexts represents a promising area for future work. Additionally, our research primarily addressed the direct binding of transcription factors to their respective sites. However, gene expression is frequently regulated through indirect mechanisms as well. For instance, they may sequester transcription factors, inhibit transcription, or displace transcription factors already bound to DNA [26], [82]. These indirect interactions can significantly impact gene expression [83], [84], particularly in producing robust responses under stochastic conditions [26], [82]. Exploring gene regulation through indirect binding mechanisms, especially in the context of spatial heterogeneity, could provide valuable insights and is another compelling direction for further investigation.

## 4 Materials and methods

### 4.1 Mathematical model describing gene regulatory networks

#### 4.1.1 One binding site gene regulatory network model

To describe the simplest case of a gene regulatory network (Fig. 1A), we used a model with one binding site on DNA with a binding state *X* ($D^{X},X\in \{0,1\}$):

**Figure:**
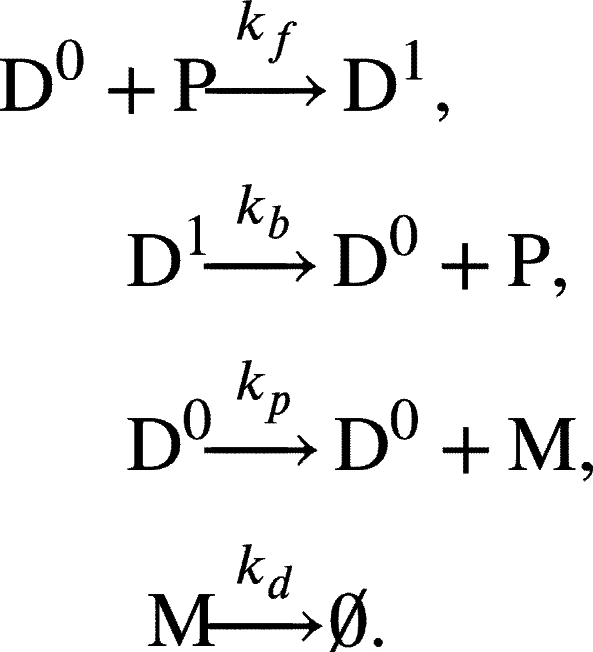

DNA with a single binding site reversibly binds to a transcriptional repressor protein (P), characterized by binding and unbinding rates $k_{f}$ and $k_{b}$, respectively. Specifically, unoccupied DNA ($D^{0}$) can bind to P to form occupied DNA ($D^{1}$). mRNA (M) is transcribed only from $D^{0}$ at a rate of $k_{p}$. M decays at a rate of $k_{d}$.

#### 4.1.2 Two binding site gene regulatory network model and its simplification

DNA with multiple binding sites exhibits more complex gene regulation compared to DNA with a single binding site [26], [68], [69]. To investigate these properties, we utilized the following model that describes the binding of P to DNA with two binding sites:(1)
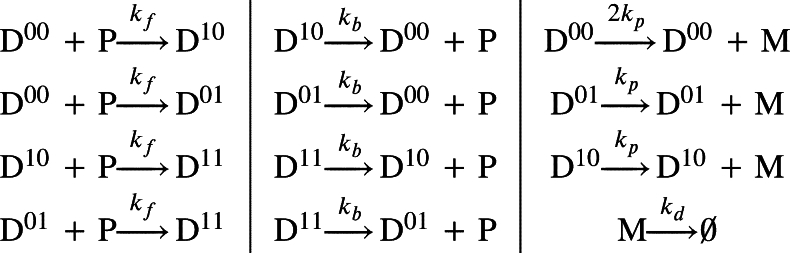


In this model, DNA with two binding sites reversibly binds to P with binding rate $k_{f}$ and unbinding rate $k_{b}$. Unoccupied DNA ($D^{00}$) can bind P at either site, forming $D^{01}$ or $D^{10}$. These singly-occupied states can then bind another P at the remaining site, forming fully occupied DNA ($D^{11}$). The transcription of M occurs at a rate of $2k_{p}$ from $D^{00}$, at a rate of $k_{p}$ from $D^{01}$ or $D^{10}$, and not at all from $D^{11}$. This mechanism can be described by the following chemical master equations (CMEs) [26]:

(2) dpm00dt=−2kfpm00P+kbpm10+kbpm01−kdmpm00+kd(m+1)pm+100=−2kppm00+2kppm−100,dpm10dt=kfpm00P−kfpm10P−kbpm10+kbpm11−kdmpm10+kd(m+1)pm+110=−kppm10+kppm−110,dpm01dt=kfpm00P−kfpm01P−kbpm01+kbpm11−kdmpm01+kd(m+1)pm+101=−kppm01+kppm−101,dpm11dt=kfpm10P+kfpm01P−2kbpm11−kdmpm11+kd(m+1)pm+111.

Here, $p_{m}^{X}$ represents the joint probability that DNA is in the state $D^{X}$ with *m* mRNAs, where X∈{00,01,10,11} denotes the DNA binding state and *P* denotes the number of repressor proteins. To model the binding and unbinding of $D^{X}$ to P, we used $k_{f}p_{m}^{X}\cdot P$ and $k_{b}p_{m}^{X}$, respectively, which describe the stochastic transitions between DNA states. Transcription is activated in the remaining DNA state at a rate $k_{p}$. For example, $p_{m}^{k}$ decreases at the rate $k_{p}p_{m}^{X}$ and increases at the rate $k_{p}p_{m-1}^{X}$. However, when both binding sites are occupied (i.e., $D^{11}$), transcription is fully repressed, meaning that mRNA transcription has no impact on the change in $p_{m}^{11}$. To represent the degradation of each mRNA, $p_{m}^{X}$ decreases at the rate $k_{d}mp_{m}^{X}$ and increases at the rate $k_{d}(m+1)p_{m+1}^{X}$.

By substituting $p_{m}^{\left(0\right)}=p_{m}^{00}$, $p_{m}^{\left(1\right)}=p_{m}^{10}+p_{m}^{01}$, and $p_{m}^{\left(2\right)}=p_{m}^{11}$, we can simplify these equations (Eq. (2)) into the following compact form:

(3) dpm(0)dt=−2kfpm(0)P+kbpm(1)−kdmpm(0)+kd(m+1)pm+1(0)−2kppm(0)=+2kppm−1(0),dpm(1)dt=2kfpm(0)P−kfpm(1)P−kbpm(1)+2kbpm(2)−kdmpm(1)=+kd(m+1)pm+1(1)−kppm(1)+kppm−1(1),dpm(2)dt=kfpm(1)P−2kbpm(2)−kdmpm(2)+kd(m+1)pm+1(2).

In other words, the gene expression depends on the number of unoccupied binding sites, rather than the specific DNA binding state. Thus, the system can be simplified with respect to the free DNA binding site (D) as follows:

**Figure:**
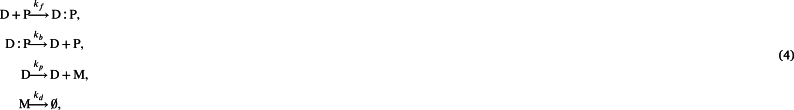

where D:P represents the complex form of D and P. Instead of simulating the complicated model, we implemented the equivalent simplified model (Eq. (4)).

### 4.2 Stochastic simulation of heterogeneous environments using compartment-based Gillespie algorithm

In order to model the gene regulation (Eq. (4)) in heterogeneous environments, we employed a spatial stochastic simulation algorithm, specifically using a compartment-based Gillespie algorithm [30]. To realize this algorithm, we first partitioned the one-dimensional domain of length *L* into *K* compartments of size h=L/K. We then simulated the diffusion between these compartments and the reaction within each compartment. Across compartments, repressor proteins can diffuse at a rate of $d=\delta /h^{2}$, where *δ* is the diffusion coefficient of the repressor protein. Specifically, the diffusion of the repressor protein can be described by the following linear reactions: , where $P_{i}$ denotes the repressor proteins in the *i*-th compartment (Note that the interpretation of the subscript here differs from that in the previous section.) Reactions within each compartment followed the dynamics described in Eq. (4), within each compartment. By denoting species X in the *i*-th compartment by $X_{i}$, and its number by $X_{i}$, the propensity functions of the stochastic reaction-diffusion equation can be written as in Table 1 [13], [30].

**Table 1:** Propensity functions of the full model describing the gene regulatory network used in the spatial SSA.

Propensity functions		Propensity
	*i* = 1,…,*K*	$\frac{k_{f}}{\Omega }D_{i}\cdot P_{i}$
	*i* = 1,…,*K*	*k*_*b*_*D*:*P*_*i*_
	*i* = 1,…,*K*	*k*_*p*_*D*_*i*_
	*i* = 1,…,*K*	*k*_*d*_*M*_*i*_
	*i* = 2,…,*K*	*dP*_*i*_
	*i* = 1,…,*K* − 1	*dP*_*i*_

For the spatial SSA of Fig. 2, Fig. 3, we set the size of the nucleus to L=10μm, which is the typical size of a mammalian nucleus [76]. Furthermore, we set *K* to 30, since using *K* larger than 30 resulted in consistent stochastic dynamics (Supplementary Fig. S5), indicating that K=30 is sufficient to capture spatial heterogeneity. This choice of *K* corresponds to each compartment size h=0.33μm. To simplify, we define Ω=1 to represent the size of a single compartment (*h*), resulting in the total system size Ω×K=30.

At the boundary compartments (i=1 and i=K), diffusion is constrained: no leftward diffusion and rightward diffusion occur at i=1 and i=K, respectively. In Fig. 2, to ensure tight binding between DNA and *P*, we set the binding and unbinding rates as $k_{f}/\Omega =5000s^{-1}$, $k_{b}=100s^{-1}$, respectively (resulting in a dissociation constant $K_{d}/\Omega =\frac{100}{5000}=0.02$) [13]. Additionally, we set $k_{p}=50s^{-1}$, consistent with the order of magnitude used in [13], and $k_{d}=0.001s^{-1}$, which falls within the typical degradation range for mRNA [76]. The simulation was conducted for $T_{max}=50s$.

To describe various cases in gene regulation, we utilized different initial conditions with specific values of $D_{i}$ and $P_{i}$ at time t=0, denoted as $D_{i}(0)$ and $P_{i}(0)$, respectively.

In Fig. 2A-D, the initial conditions were set such that DNA with one binding site was immobile at the center of the nucleus, and P was uniformly distributed within the nucleus: $D_{15}(0)=1$, $D_{i}(0)=0$ for i≠15, and $P_{i}(0)=1$ for i=1,…,K.

In Fig. 2A-B, P was allowed to diffuse quickly between compartments at a rate of $d=180.4s^{-1}$, corresponding to a diffusion coefficient of $\delta =20\;{\mu \text{m}}^{2}/s$, which is within the typical range for protein diffusion coefficients [85]. By contrast, in Fig. 2C-H and Fig. 3C-D, the diffusion rate was significantly slowed to $d=1.8s^{-1}$, corresponding to the diffusion coefficient of the PER2 protein, $\delta =0.2\;{\mu \text{m}}^{2}/s$ [86].

In Figs. 2E and 3C, the initial distribution of P was modified to create a nonuniform distribution within the nucleus, as opposed to the uniform distribution in Fig. 2C. Specifically, $P_{i}(0)=1$ for i=11,…,20, and $P_{i}(0)=5$ for i∉11,…,20.

For Figs. 2G and 3D, the initial conditions for D were altered while maintaining the initial conditions for P from Fig. 2C. In this case, DNA with two binding sites was immobile at the center of the nucleus, with $D_{15}(0)=2$ and $D_{i}(0)=0$ for i≠15.

In Supplementary Fig. S2, we simulated a slightly modified version of Eq. (4) to model activating gene regulation with the spatial SSA. In this model, M is transcribed from the complex D:P, rather than from D alone, with the rate of $k_{p}$. For this simulation, parameters were set as $k_{f}/\Omega =700s^{-1}$ and $k_{b}=900s^{-1}$, with all other parameters identical to those in Fig. 2. The same initial conditions as those in Fig. 2 were used.

In Supplementary Fig. S4, we expanded the model to a two-dimensional domain by adapting the simulation to a 30×30 grid representing a 10μm×10μm area. Two-dimensional diffusion was implemented consistently to the one-dimensional diffusion in Table 1. *D* was set to 1 within the (15,15) grid cell and *P* was set to 1 for every grid cell. Other parameters were identical to those used in Fig. 2.

### 4.3 Stochastic simulation of homogeneous environments using Gillespie algorithm

To simulate gene regulation in homogeneous environments, we utilized the stochastic simulation algorithm (SSA), which is also known as the Gillespie algorithm [87]. Different from the spatial SSA, the SSA ignores spatial heterogeneity. For example, P can bind to D at any location within the nucleus. In contrast, the spatial SSA model restricts P to binding with D only when they are sufficiently close to each other. Thus, we calculated the propensity functions for the SSA as if all the molecules are in a single compartment as described in Table 2.

**Table 2:**
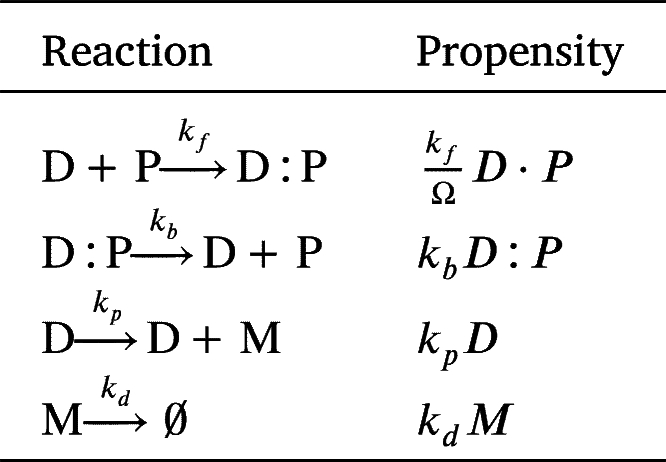
Propensity functions of the full model describing the gene regulatory network used in the SSA.

To match the volume of the whole system size in the spatial SSA, Ω was set to K=30. In addition, for Fig. 2, we utilized the same rate constants as in the spatial SSA. Initially, the number of D and P, *D* and *P*, were set to the following initial conditions were used: [D(0),P(0)]=[1,30] for Fig. 2A and C, [D(0),P(0)]=[1,110] for Fig. 2E, and [D(0),P(0)]=[2,30] for Fig. 2G.

Initially, the number of D and P, denoted as *D* and *P*, were set as follows: [D(0),P(0)]=[1,30] for Fig. 2A and C, [D(0),P(0)]=[1,110] for Fig. 2E, and [D(0),P(0)]=[2,30] for Fig. 2G.

In Supplementary Fig. S2, analogous to the spatial SSA, we simulated the model for activating gene regulation with the SSA. Here, the transcription of M occurs from the complex D:P instead of from D alone, with a transcription rate of $k_{p}$. For this simulation, the parameters were set as $k_{f}/\Omega =700s^{-1}$ and $k_{b}=900s^{-1}$, with all other parameters identical to those in Fig. 2. The initial conditions were the same as those used in Fig. 2.

### 4.4 Derivation of two reduction model equations: slQSSA and stQSSA

Although the spatial SSA is required to describe gene regulation with spatial heterogeneity, it is significantly more computationally expensive than the traditional SSA. To address this issue, we applied the QSSA, which approximates the amount of free DNA that rapidly reaches a quasi-steady state (QSS) due to the fast reaction, thereby allowing us to focus only on the slow reactions. To apply QSSA to stochastic systems, it is necessary to solve the CMEs to obtain the average number of stationary states. While this is typically challenging, it can be computed analytically when the number of binding sites is low. Specifically, if we have $D_{i}$ binding sites and $P_{i}$ protein molecules at *i*-th compartment, the average number of the free binding sites can be calculated as the following [13]:

(5) $$\begin{array}{cc} \langle D\rangle & =\left(\sum_{l=D_{0,i}}^{D_{i}}\frac{lK_{d}^{l}}{l!(D_{i}-l)!(P_{i}-D_{i}+l)!}\right)\\ & \;\cdot {\left(\sum_{l=D_{0,i}}^{D_{i}}\frac{K_{d}^{l}}{l!(D_{i}-l)!(P_{i}-D_{i}+l)!}\right)}^{-1}, \end{array}$$

where $D_{0,i}=\max\{0,D_{i}-P_{i}\}$. This approach is known as the slQSSA [13]. In our study, we applied the slQSSA within the spatial SSA framework, considering three states (i.e., $D_{i}\leq 3$) based on the number of occupied binding sites as follows:

(6) $$\begin{array}{c} D_{lq,i}=\left\{ \begin{array}{cc} \frac{(P_{i}-D_{i}+2)D_{i}K_{d}+D_{i}(D_{i}-1)K_{d}^{2}}{2(P_{i}-D_{i}+2)(P_{i}-D_{i}+1)+2(P_{i}-D_{i}+2)D_{i}K_{d}+D_{i}(D_{i}-1)K_{d}^{2}}, & D_{i}\leq P_{i},\\ \frac{(P_{i}-D_{i}+2)(D_{i}-P_{i}+1)P_{i}K_{d}+P_{i}(P_{i}-1)K_{d}^{2}/2}{2(D_{i}-P_{i}+2)(D_{i}-P_{i}+1)+2(D_{i}-P_{i}+2)P_{i}K_{d}+P_{i}(P_{i}-1)K_{d}^{2}}, & D_{i}>P_{i}, \end{array}\right.\\ (i=1,...,K). \end{array}$$

As the number of binding sites increases, the slQSSA equation (Eq. (6)) becomes more complex, making it more difficult to interpret. As an alternative, previous studies have used the stQSSA [9], [10], [11], [12], [13], [52], [54], [55], [56], [57], [58], [59], [60], [61]. The stQSSA $D_{tq,i}$ can be derived using the concentration-based free DNA obtained from the deterministic tQSSA:

(7) $$\begin{array}{c} D_{tq,i}=\{D_{i}-P_{i}-K_{d}-\sqrt{{\left(D_{i}-P_{i}-K_{d}\right)}^{2}+4D_{i}K_{d}}\}/2,\;(i=1,...,K). \end{array}$$

While it has been believed that the stQSSA accurately captures stochastic dynamics at a low computational cost [9], [10], [11], [12], [13], [52], [54], [55], [56], [57], [58], [59], [60], [61], recent research showed that the stQSSA can be inaccurate when two species tightly bind and their molar ratio is ∼1:1 [13]. Based on the obtained $D_{lq,i}$ and $D_{tq,i}$, we calculated the propensity functions to verify that the two reduced models accurately approximate the full model. (See Table 3.)

**Table 3:**
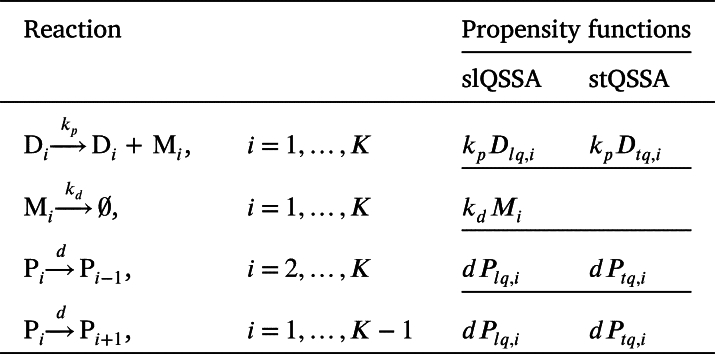
Propensity functions of the reduced (stQSSA and slQSSA) models describing the gene regulatory network used in the spatial SSA.

To calculate the diffusion propensities, we obtained the number of the free repressor proteins in the *i*-th compartment using the slQSSA and stQSSA, denoted as $P_{lq,i}$ and $P_{tq,i}$, respectively. This is because bound repressor proteins cannot diffuse, and thus the number of free repressor proteins should be predicted by using the slQSSA or stQSSA. Specifically, the number of free repressor proteins is calculated by subtracting the number of bound *P* ($D:P_{lq,i}$ or $D:P_{tq,i}$) from the total *P* obtained from the simulation ($P_{i}$), using $P_{lq,i}=P_{i}-D:P_{lq,i}$, $P_{tq,i}=P_{i}-D:P_{tq,i}$, where $D:P_{lq,i}=D_{i}-D_{lq,i}$, $D:P_{tq,i}=D_{i}-D_{tq,i}$.

To simulate two reduction models (slQSSA and stQSSA) in Fig. 3, we set *K* to 30 and divided the 1-dimensional domain [0,10] into K=30 compartments of length h=0.33μm. We used Ω=1, $k_{f}/\Omega =5,000s^{-1}$, $k_{b}=100s^{-1}$, $k_{p}=50s^{-1}$, and $k_{d}=0.001s^{-1}$. The diffusion rate was set to $d=0.01s^{-1}$, and the simulation ran for $T_{max}=50s$. For Fig. 3C-D, *P* was set to diffuse slowly between compartments at a rate of $d=0.018s^{-1}$. In Fig. 3C-D, the QSSA models use the same initial conditions as the full model.

### 4.5 Stochastic simulation of molecular oscillation

We constructed a gene regulatory model with a negative feedback loop to investigate the periodicity of biological oscillations across three models [38], [74], [83], [88], [89], [90]: the full, the slQSSA, and the stQSSA. Unlike in the previous model, this domain represents the entire cell. Specifically, the cell is 30μm in size, with a 10μm region at the center representing the nucleus, which is the typical size of mammalian cells [76]. We assumed that the DNA is immobile within the nucleus, while repressor proteins can diffuse in and out of the nucleus. Additionally, we assumed that proteins produced within the cell are translocated to the periphery immediately for simplicity. Based on these assumptions, the propensity functions for the oscillation model are presented in Table 4.

**Table 4:**
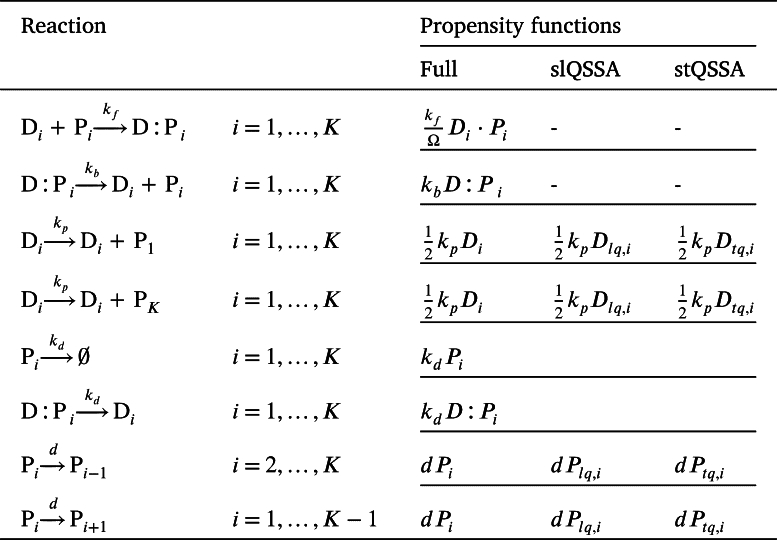
Propensity functions of the full and reduced (stQSSA and slQSSA) models describing biological oscillator used in the spatial SSA.

For Fig. 4, we used K=30 and thus h=1μm, and we assumed that $d=1.8s^{-1}$ as in Fig. 2C-H. We set Ω=1, and the rate constants were set to $k_{f}/\Omega =10,000s^{-1}$, $k_{b}=100s^{-1}$ to ensure tight binding ($K_{d}=0.02$), and $k_{p}=10s^{-1}$, $k_{d}=0.01s^{-1}$. The simulation ran for a total duration of $T_{max}=5000s$. For the initial conditions, DNA with two binding sites was assumed to be immobile in the center of the nucleus, and repressor proteins had a uniform distribution in the nucleus: $D_{15}(0)=2$, $D_{i}(0)=0$ for i≠15, and $P_{i}(0)=1$ for i=1…K.

### 4.6 Quantifying the period of molecular oscillations

In Fig. 4D, we quantified the period of oscillation of *P* by utilizing the decaying cosine function following previous studies [73], [27], [74]. First, we calculated the autocorrelation function C(s) of the total protein concentration in the cell over time, P(t), using the following formula: $C(s)=\frac{\int \left[P(t+s)-\overset{¯}{P}\right]\left[P(t)-\overset{¯}{P}\right]dt}{\int {\left[P(t)-\overset{¯}{P}\right]}^{2}dt}$, where $\overset{¯}{P}$ is the time average of P(t). We then fitted this to a decaying cosine function $\overset{˜}{C}(s)=\exp(-s/\tau )\cos(2\pi s/T)$ to estimate the period of oscillation *T*, where the correlation time *τ* describes how fast the C(s) exponentially decays. To ensure accuracy, the period was calculated using the simulation data from the last 2,500*s* of a 5,000*s* run, with the first 2,500*s* excluded to eliminate the influence of transient dynamics. To obtain the period distribution in Fig. 4D, we independently calculated the period of 1,000 iterative simulations.

## CRediT authorship contribution statement

**Seok Joo Chae:** Writing – review & editing, Writing – original draft, Visualization, Validation, Software, Methodology, Investigation, Formal analysis, Data curation. **Seolah Shin:** Writing – review & editing, Writing – original draft, Visualization, Validation, Software, Methodology, Investigation, Formal analysis, Data curation. **Kangmin Lee:** Investigation, Software, Writing – review & editing. **Seunggyu Lee:** Writing – review & editing, Writing – original draft, Visualization, Validation, Supervision, Resources, Project administration, Methodology, Investigation, Funding acquisition, Formal analysis, Conceptualization. **Jae Kyoung Kim:** Writing – review & editing, Writing – original draft, Visualization, Validation, Supervision, Resources, Project administration, Methodology, Investigation, Funding acquisition, Formal analysis, Conceptualization.

## Declaration of Competing Interest

The authors declare that they have no known competing financial interests or personal relationships that could have appeared to influence the work reported in this paper.
